# Diagnostic Procedures and Management of Dry Eye

**DOI:** 10.1155/2013/309723

**Published:** 2013-08-19

**Authors:** Snježana Kaštelan, Martina Tomić, Jasminka Salopek-Rabatić, Branko Novak

**Affiliations:** ^1^Department of Ophthalmology, Clinical Hospital Dubrava, Avenija Gojka Šuška 6, 10000 Zagreb, Croatia; ^2^Department of Ophthalmology, University Clinic Vuk Vrhovac, Clinical Hospital Merkur, Zajčeva ulica 19, 10000 Zagreb, Croatia

## Abstract

Dry eye disease or dysfunctional tear syndrome is among the most frequent diagnoses in ophthalmology. It is a multifactorial disease of the ocular surface and tear film which results in ocular discomfort, visual disturbances, and tear instability with potential damage to the cornea and conjunctiva. Risk factors for dry eye syndrome include age, sex (female gender), race, contact lens wear, environment with low humidity, systemic medications, and autoimmune disorders. The aim of this paper is to present the systematic classification, epidemiology, diagnostic procedures, and advances in the management of dry eye disease. The recent improvements in comprehending the underlying etiologic factors will inevitably improve future classifications and diagnostic abilities leading to more effective therapeutic options. Treatment of this highly prevalent condition can drastically improve the quality of life of individuals and prevent damage to the ocular surface.

## 1. Introduction

Dry eye syndrome is recognized as a growing public health problem and one of the most frequent reasons for seeking ophthalmological intervention. Various terms have been used to describe dry eye disease (DED) including keratoconjunctivitis sicca and, more recently, dysfunctional tear syndrome suggesting that the name more accurately reflects pathophysiological changes. The definition of DED which includes etiology, pathophysiology, and symptoms was recently improved in the light of new findings about the role of tear hyperosmolarity and ocular surface inflammation in dry eye and its effect on visual function [1]. According to current knowledge dry eye can be defined as a multifactorial disease of the tears and ocular surface that results in symptoms of discomfort, visual disturbance, and tear film instability, with potential damage to the ocular surface. It is accompanied by increased osmolarity of the tear film and inflammation of the ocular surface [1, 2]. 

## 2. Classification of Dry Eye 

Dry eye is a condition that results in dryness of the conjunctiva and cornea due to decreased tear function of tear glands or rapid evaporation of tears. On the basis of these underlying pathologic processes dry eye disease could be classified as tear deficiency or hyposecretive dry eye which includes Sjögren's syndrome and non-Sjögren's tear deficiency and evaporative or hyperevaporative dry eye (Table 1) [1–4]. This classification often neglects patients with simultaneous occurrence of hyperevaporation and hyposecretion. 

The term “tear-deficient dry eye” implies that this condition is caused by the lacrimal acinar destruction or dysfunction with reduced lacrimal tear secretion and volume. This in turn causes tear hyperosmolarity, since water evaporates from a reduced aqueous tear pool. Tear film hyperosmolarity causes hyperosmolarity of the ocular surface epithelial cells which stimulates a cascade of inflammatory events [1, 2]. 

Aqueous-deficient dry eye has two major groupings: Sjögren's syndrome and non-Sjögren's syndrome dry eye. Sjögren's syndrome is an exocrinopathy in which the lacrimal and salivary glands as well as other organs are affected by autoimmune processes and can be divided into two subgroups: primary and secondary Sjögren's syndrome. Conversely non-Sjögren's syndrome is a form of tear deficient dry eye due to lacrimal dysfunction, where the systemic autoimmune characteristic of Sjögren's syndrome has been excluded. The most common form is age-related dry eye [3, 4]. 

Evaporative dry eye may be intrinsic as a result of meibomian lipid deficiency, poor lid congruity and lid dynamics, low blink rate, and the effects of drug use. Extrinsic evaporative dry eye embraces those etiologies that increase evaporation including vitamin A deficiency, the action of toxic topical agents such as preservatives (benzalkonium chloride), and topical anesthesia. Patient wearing contact lenses is more prone to have dry eye symptoms. Disease of the exposed ocular surface including allergic eye disease may lead to destabilization of the tear film and add a dry eye component to the ocular surface [1, 2, 5, 6]. 

## 3. Prevalence

The prevalence of dry eye symptoms increases with age and has been reported in approximately 5% to 30% of the study population depending on the criteria used to define the condition and the differences in the definition of the study population [5, 7–11]. Problems encountered in the exact estimation of prevalence may rely on whether the data came from general population surveys or physician assessments. Among patients diagnosed by physicians, estimated prevalence may vary depending on the diagnostic criteria used and the clinicians' subjective assessments [12]. 

In addition to age, the risk factors for development of dry eye include race or ethnicity; greater incidence is seen in patients of Chinese, Hispanic, Asian, and Pacific Islands descent and female sex (women report dry eye twice as often as men). Women are particularly susceptible to dry eye symptoms, especially those receiving estrogen replacement therapy [7]. The prevalence of dry eye is higher in the presence of ocular conditions such as blepharitis, meibomian gland dysfunction, and conjunctival disease; in the presence of systemic conditions including arthritis, osteoporosis, gout, and thyroid disorders; and after corneal, retinal, or ocular oncologic surgery [12, 13]. 

## 4. Etiology 

The health of the ocular surface is maintained by efficient production, secretion, and elimination of a physiologically stable tear film. The tear film has traditionally been considered to consist of three distinct layers: a thin outer lipid layer that is secreted by the meibomian glands, an inner layer of mucous secreted by goblet cells of the conjunctiva, and a complex middle aqueous layer secreted by the main lacrimal and accessory gland that contains a wide array of dissolved substances. A newer concept describes the tear film as a dynamic mucinous gel that decreases in density toward the outer layer. The tear film maintains the structure and functioning of the cornea under normal physiological conditions in individuals with normal ocular anatomy. The tear film maintains an optically uniform surface, lubricates and nourishes eye tissue, washes out cellular debris and foreign bodies, and also protects from bacterial infections [14–16]. 

Inflammation is a central feature of ocular surface disease. An association between ocular symptoms and activation of T lymphocytes has been established in patients with Sjögren's syndrome [3]. Today it is understood that a local autoimmune occurrence could appear irrespective of systemic autoimmune disease. Conjunctival inflammation is manifested by infiltration of inflammatory cells and upregulated expression of immune markers. Hyperosmolar stress has proinflammatory effect. A better understanding of the immunopathological mechanisms of ocular surface disorders etiology corresponds with modification of applied therapy [15, 16]. 

## 5. Risk Factors 

As previously noted, risk factors for dry eye include female sex, older age, postmenopausal estrogen therapy, computer use, contact lens wear, a diet low in omega-3 essential fatty acids or a high ratio of omega-6 to omega-3 fatty acids, refractive surgery, vitamin A deficiency, radiation therapy, bone marrow transplantation, hepatitis C, and certain classes of systemic and ocular medications, including antihistamines. Vitamin A deficiency is a well-recognized risk factor for dry eye, and the etiology includes inadequate intake due to alcohol-related nutritional deficiency, stomach surgery, malabsorption, eating disorders, and a vegan diet. 

Other risk factors include diabetes mellitus, human immunodeficiency virus, HIV and human T cell lymphotropic virus-1 infection, connective tissue diseases, systemic cancer chemotherapy, and medications such as isotretinoin, antidepressants, anxiolytics, beta-blockers, and diuretics. However, a comprehensive study of these factors is still lacking. Conflicting results have been reported on the association between dry eye and some factors, including alcohol, cigarette smoking, caffeine, acne, and menopausal status. Likewise, very few reports exist on the risk of dry eye with use of oral contraceptives and during pregnancy [5, 9, 10, 17–19]. 

## 6. Symptoms

It is often incorrectly assumed that symptoms of dry eye are the main feature of this disease, whereas unfortunately they do not always correspond with diagnostic test results except in severe cases. The symptoms that patients describe are the same ocular sensations felt in other ocular surface disorders, namely, reports of a gritty, sandy foreign body sensation and visual disturbances. Visual complaints are highly prevalent among dry eye patients usually described as blurry vision that clears temporarily upon blinking [6]. These transient changes, resulting from disrupted tear film in the central cornea, can be profound with marked drops in contrast sensitivity and visual acuity thereby affecting workplace productivity and vision-related quality of life [6, 19]. 

## 7. Diagnostic Procedures

The diagnosis of ocular surface disease is based on the patient's symptoms and medical history which should include questions about topical and systemic medications used and possible exposure to aggravating factors. Currently available diagnostic tests and external examinations are also indispensable for every practitioner in order to reach the decision on the most suitable treatment [1]. Symptom questionnaires allow for rapid and efficient collection of relevant information and can facilitate diagnosis of ocular surface disorders. Questionnaires and dry eye index scores can be useful to detect the presence of dry eye and to evaluate the effect of therapeutic treatment. Several questionnaires are available, with the most common being the Ocular Surface Disease Index (OSDI) [20]. However, there is still no standardized dry eye disease questionnaire that is universally accepted. After patient's medical history is obtained and questionnaires administered, clinical examination of the anterior segment and objective tests are necessary to confirm the diagnosis of dry eye [2, 21].

### 7.1. Objective Testing

Objective tests for dry eye can be divided into tests that examine the tears and those that examine the integrity of the ocular surface. The former can further be subdivided into tests that investigate the quantity, quality, or functional properties of tears. 

### 7.2. Tear Quality

Some authors consider that the determination of tear osmolarity is significant in dry eye diagnosis; however, it requires expert technical support, and its use has to date been confined to specialized laboratories [22]. The appearance of new more affordable osmometers has expanded their use in everyday practice [23]. The most common test for determining tear film quality in use today is the tear breakup time (TBUT) which is described later in this paper. 

### 7.3. Tear Quantity

 The most widely used technique to evaluate tear quantity is the Schirmer test 1, performed without anesthesia. In this test, a 5 × 35 mm strip of filter paper that is bent 5 mm from the end is placed under the lower eyelid on the temporal side. The strip is kept in place for 5 minutes and then the length of the moistened strip is measured. A result yielding less than 5 mm shows aqueous tear deficiency. Insertion of the strip for 5 minutes may cause discomfort with reflex tears secretion. Therefore, as an alternative, some practitioners keep the paper in place for 2 minutes or apply a topical anesthetic prior to placing the strip (Schirmer II) [14, 24]. Another noninvasive method used is the tear meniscus height measurement on the lower eyelid, whereby a height lower than 0.2 mm is associated with tear deficiency [25]. 

### 7.4. Stains and Dyes of the Ocular Surface

 Fluorescein is useful in assessing dry eye where its application can determine the integrity of the corneal and conjunctival epithelium. The normal epithelium does not stain; however, when the mucous layer is absent, the dye penetrates and stains the epithelium. Evaluation after 2 minutes is recommended since premature examination of the surface may underestimate the degree of damage [2, 14]. Lissamine green is another dye used to evaluate the anterior segment and is used to stain dead or degenerated cells and produces less irritation compared with rose bengal dye. Grading ocular surface staining after application of vital dyes is a central component of dry eye diagnosis [26]. Fluorescein is also used for classic tear film stability tests. Fluorescein is applied into the lower fornix, and the patient is first asked to blink several times and then to avoid blinking. A broad slit-lamp beam with cobalt blue filter is used to scan the tear film. The presence of black spots or lines indicates the appearance of dry spots in the tear film [27]. Tear film breakup time (TBUT) is the interval between the blink and the appearance of the first randomly distributed dry spot. A TBUT of less than 10 seconds is considered abnormal [14, 27]. 

### 7.5. Additional Tests

Patients with keratoconjunctivitis sicca often have a decreased eyelid blink rate as result of diminished corneal sensitivity due to ocular surface inflammation. However, reduced corneal sensation is also observed after refractive surgery as with normal aging [28]. The ocular protection index (OPI) was designed in an attempt to provide a combined measurement of tear film instability and the interblink interval (IBI). It is calculated by dividing the number of eyelid blinks in 1 minute by 60 whereby the normal IBI is between 10 and 12 seconds. Dividing the TBUT value by the IBI, the OPI value is obtained. OPI values less than 1 suggest that the tear film destabilizes between blinks whilst OPI values of 1 or higher seem to correlate with patient symptoms [29]. Additional useful tests include conjunctival impression cytology (to evaluate the goblet cells), brush cytology (to analyze the possible inflammation of the ocular surface), and measuring the quantities of lysozyme and lactoferrin in the tears. Decreases in the concentration of these two major lacrimal proteins secreted by the lacrimal glands in tear film indicate lacrimal gland dysfunction [30]. 

### 7.6. Emerging Technologies

Newer research attempts to detect and develop new diagnostic technologies that will show promise for advancing our ability to investigate, monitor, and diagnose dry eye disease in the future. There is particular interest in noninvasive or minimally invasive technologies, namely, various instruments that can detect optical changes in tear film consistency without touching the eye that could be adapted for everyday clinical use. Research is continuously striving to develop and improve technologies that allow changes in tears at the ocular surface to be detected while causing the least disturbance to tear film dynamics during sampling [31]. 

## 8. Complications of Untreated Dry Eye

Since tears protect the ocular surface from infection in severe cases of untreated dry eye syndrome, the associated inflammation can damage the conjunctiva and the cornea with an increased risk of eye infection. Fortunately, most cases of dry eye related conjunctivitis are mild and do not need specific treatment. If inflammation however becomes severe and chronic, timely and appropriate therapy must be applied prior to damages of the corneal surface which leads to irreparable ulceration or scarring. These complications can produce more severe symptoms such as extreme sensitivity to light, pain, red eyes, and loss of vision [14, 32]. 

## 9. Treatment 

The prime goal of treatment of the ocular surface disorders includes relief of symptoms, improvement of visual acuity and quality of life, restoration of ocular surface and tear film, and correction of underlying defects. Treatment options comprise of hygiene and life style changes, artificial or autologous serum tear use, and anti-inflammatory drug therapy, as well as physical and surgical procedures to increase tear retention. Treatment should be adjusted to incorporate the patient's response and must maintain a balance between efficacy, safety, and patient convenience [14]. 

The simplest and most effective way to relieve symptoms of dry eye is a lifestyle change. Patients should be advised to avoid long exposure to computers, TV, and reading which is associated with a reduced blink rate and thus increased evaporation. The use of artificial tears and short breaks during these activities are recommended. Humidification of air in the home and work place could also alleviate undesirable effects. Avoidance of hot, windy, low-humidity, and high-altitude environments as well as smog and smoke is also advisable [14, 33]. 

Eyelid hygiene, warm compresses, and topical antibiotics when needed are essential for chronic blepharitis and meibomian gland dysfunction treatment which can be associated with tear dysfunction. These measures reduce bacterial induced changes in the lipid component of the tear film, which in turn reduces evaporative tear loss [33]. 

It has been shown that a higher dietary intake of omega-3 fatty acids with lower dietary ratio of omega-6 to omega-3 fatty acids as well as use of supplements containing linoleic and gamma-linoleic acid decreases the risk associated with dry eye symptoms [34, 35]. 

Tear supplements provide only temporary relief of dry eye symptoms and usually contain preservatives which can irritate the eye and additionally exacerbate symptoms. Thus patients requiring tear supplements more than 4 times a day should be prescribed preservative-free products. Artificial tears cannot replace the cytokines and growth factors which are comprised in normal tears and produced by normal-functioning lacrimal glands and thus do not have direct anti-inflammatory effect [14, 33]. 

Keeping in mind that inflammation is a key component of the pathogenesis of dry eye, the efficacy of some anti-inflammatory agents for dry eye disease treatment has been investigated. This form of therapy may be used for patients who have corneal disease and have persistent symptoms despite extensive use of artificial tears. The most widely used anti-inflammatory agents are topical corticosteroids, tetracyclines, cyclosporine A, and in some cases in patient with Sjögren's syndrome pilocarpine. Before using this medication possible side effects should be assessed with respect to their potential benefit [14, 15, 33]. 

Autologous serum tears is the fluid component of full blood that remains after clotting and contains fibronectin, vitamin A, cytokines, growth factors, and anti-inflammatory substances. Kojima et al. observed the benefit of 20% autologous serum solution showing symptom relief, improvement of TBUT test, and rose-bengal staining score in patients with dry eye disease. It should however be noted that this kind of treatment should be reserved for management of severe cases only [36]. Kinoshita et al. in their randomized, multicenter phase 3 study showed that administration of 2% rebamipide was effective in improving both the objective signs and subjective symptoms of dry eye [37]. Those findings, in addition to the well-tolerated profile of 2% rebamipide, clearly showed that it is also an effective therapeutic method for dry eye.

Punctual plugs relieve dry eye symptoms in patients with Sjögren's syndrome, filamentary keratitis, chronic Stevens-Johnson syndrome, trachoma, neurotrophic and diabetic keratopathy, keratitis sicca, and in patients with dry eye after refractive surgery. Obstruction or inflammations of the lacrimal canaliculi or ducts as well as active blepharitis is a contraindication for their application. Permanent surgical punctual occlusion is an alternative to use of punctal plugs [38]. 

Moisture-retaining eye wear protects the eyes from environmental drying and increases periocular humidity [39]. Hydrophilic bandage contact lenses may be considered for corneal surface protection or pain relief or as an aid to corneal reepithelization. The lenses act as a reservoir for sustained hydration, serve as a barrier that protects the traumatized cornea, and provide splitting effect for corneal healing [40]. 

Surgical procedures suitable for treatment of severe dry eye include lid procedures (permanent punctal closure usually with cautery and tarsorrhaphy) and conjunctival procedures (conjunctival transplantation, amniotic membrane transplant, free conjunctival graft, and stem cell replacement). 

To surmise, the recommendations of the Dry Eye Workshop based on disease severity consist of four levels of treatment [14, 33].

*Level 1 (dry sensation, burning).* Education and environmental/dietary modifications; elimination of offending systemic medications; use of preserved artificial tear substitutes, gels, and ointments; and eyelid therapy.

*Level 2 (itching, pain, photophobia). *Use of nonpreserved artificial tear substitutes; anti-inflammatory agents (topical corticosteroids, topical cyclosporine A, topical or systemic omega-3 fatty acids); tetracyclines (for meibomianitis or rosacea); punctal plugs (after the inflammation has been brought under control); secretagogues (pilocarpine); and moisture chamber spectacles. 

*Level 3 (red eyes, foreign body sensation, pain, blurred vision)*. Application of autologous serum or umbilical cord serum; prescription of contact lenses; or permanent punctal occlusion. 

*Level 4 (blepharospasm, risk of corneal perforation). *Prescription of systemic antiinflammatory agents and surgery (lid surgery, tarsorrhaphy, mucous membrane grafting, salivary gland duct transposition, amniotic membrane transplantation). 

## 10. Conclusion 

Dry eye is a multifactorial disease of the tears and ocular surface with symptoms that often fail to correspond to diagnostic testing. It is a widespread problem that may often be overlooked since it is not a common cause of permanent visual morbidity [8, 16]. However, newer concepts suggest that dry eye syndrome can have a significant impact on visual function diminishing the everyday quality of life [19]. If left untreated, the patient may experience not only discomfort and visual disturbances but also ocular inflammation and scarring of the corneal surface with permanent damage [16, 19]. Management of ocular surface disorders requires thorough history and ophthalmological examination. The incorporation of a questionnaire may facilitate the evaluation of patients and aid in setting a diagnosis. A variety of treatment modalities are currently available and the selection of treatment can be simplified by classifying symptoms on a continuum from mild to severe and thereby choosing therapies that target the underlying inflammatory process with the goal of restoring the normal tear film and function. New research attempts to detect and develop new and promising diagnostic technologies that will further advance our ability to investigate, diagnose, and treat dry eye disease in the future. 

## Figures and Tables

**Table 1:** Classification of dry eye.

Dry eye			
Tear deficient (hyposecretive)		Evaporative (hyperevaporative)	
Sjoergen's syndrome	Non-Sjögren's tear deficiency	Intrinsic	Extrinsic
Primary	Lacrimal disease/deficiency	Oil deficient	Topical drug preservatives
Secondary	Lacrimal obstruction	Lid related	Vitamin A deficiency
	Reflex block	Low blink rate	Contact lens related
			Ocular surface change
			Drug related
